# Toxicity of Superparamagnetic Iron Oxide Nanoparticles on Green Alga *Chlorella vulgaris*

**DOI:** 10.1155/2013/647974

**Published:** 2013-12-04

**Authors:** Lotfi Barhoumi, David Dewez

**Affiliations:** ^1^Département de Chimie, Université du Québec à Montréal, CP 8888, Succursale Centre-Ville, Montréal, QC, Canada H3C 3P8; ^2^Laboratoire de Physiologie Intégrée, Faculté des Sciences de Bizerte, Université de Carthage, Zarzouna 7021, Tunisia

## Abstract

Toxicity of superparamagnetic iron oxide nanoparticles (SPION) was investigated on *Chlorella vulgaris* cells exposed during 72 hours to Fe_3_O_4_ (SPION-1), Co_0.2_Zn_0.8_Fe_2_O_4_ (SPION-2), or Co_0.5_Zn_0.5_Fe_2_O_4_ (SPION-3) to a range of concentrations from 12.5 to 400 **μ**g mL^−1^. Under these treatments, toxicity impact was indicated by the deterioration of photochemical activities of photosynthesis, the induction of oxidative stress, and the inhibition of cell division rate. In comparison to SPION-2 and -3, exposure to SPION-1 caused the highest toxic effects on cellular division due to a stronger production of reactive oxygen species and deterioration of photochemical activity of Photosystem II. This study showed the potential source of toxicity for three SPION suspensions, having different chemical compositions, estimated by the change of different biomarkers. In this toxicological investigation, algal model *C. vulgaris* demonstrated to be a valuable bioindicator of SPION toxicity.

## 1. Introduction

Due to their physicochemical features, superparamagnetic iron oxide nanoparticles (SPION) are widely used in medical applications such as contrast agents for magnetic resonance imaging and heating mediators for cancer therapy [1, 2]. A recent review of SPION-induced toxicity studies at cellular level in animal and human cells indicated that SPION can penetrate the cellular system by both passive diffusion and endocytosis, causing several toxic effects through the alteration of genes expressions and the generation of oxidative radicals [3]. However, their production and extensive use may contaminate aquatic environments via wastewater input, representing a risk of toxicity for different freshwater organisms. Besides endocrine disruptor and pharmaceuticals, metallic engineered nanoparticles represent one of the most important hazardous materials altering freshwater qualities. Many studies showed that the toxicity of metallic engineered nanoparticles was directly related to their surface chemistry, hydrodynamic size, chemical composition, and solubility in aqueous solution [4, 5]. A recent study demonstrated that SPION have antibacterial properties with the ability to alter metabolic functions at a higher efficiency than antibiotics or metals salts [6]. Therefore, it is of high importance to determine the toxicity potential at cellular level of hazardous metallic nanomaterials in relation to their uptake by aquatic microorganisms. In aquatic environments, green algae represent the main source of biomass production essential for animals of higher ecological trophic levels. As being able to bioaccumulate metallic contaminants, algae can be used as a bioindicator of aquatic ecosystem health. However, bioaccumulation effects of SPION on algae have been poorly examined, and such toxicological studies were mostly done on terrestrial plant species grown hydroponically [7, 8]. Currently, there is only one study concerning the toxic effects of Fe_3_O_4_ nanoparticles (35 nm) on the green alga *Chlorella vulgaris* treated 72 h to a nominal concentration range from 200 to 1600 *μ*g mL^−1^. In this study, authors showed an induction of oxidative stress and an alteration of photosynthetic activity based on absorbed CO_2_ fixation [9]. Therefore, a more in-depth toxicological investigation needs to be performed to better characterise the toxicity of SPION on the cell physiology of green algae.

In the present study, the green alga *Chlorella vulgaris* was used as a unicellular plant model organism for the toxicity characterisation of Fe_3_O_4_ (SPION-1), Co_0.2_Zn_0.8_Fe_2_O_4_ (SPION-2), and Co_0.5_Zn_0.5_Fe_2_O_4_ (SPION-3). Algal cells were exposed during 24, 48, and 72 hours in order to evaluate the evolution of SPION toxicity impact on the entire cellular system by evaluating the change in photochemical reactions of photosynthesis, cell division, and the induction of oxidative stress. This work permitted determining the risk of SPION toxicity on the viability of green algae and therefore the potential use of this algal species in a bioassay of SPION toxicity.

## 2. Material and Methods

### 2.1. Biological Material

The freshwater green alga *Chlorella vulgaris* was obtained from the *Canadian Phycological Culture Centre* (CPCC, University of Waterloo, ON, Canada). Algal culture was prepared in sterile BG-11 liquid growth medium (pH = 7; ionic strength = 0.0201) having the following final concentrations of salts composition [10]: 1.5 g L^−1^ of NaNO_3_, 0.04 g L^−1^ of K_2_HPO_4_·3H_2_O, 0.075 g L^−1^ of MgSO_4_·7H_2_O, 0.036 g L^−1^ of CaCl_2_·2H_2_O, 6 × 10^−3^ g L^−1^ of C_6_H_8_O_7_ (citric acid), 6 × 10^−3^ g L^−1^ of C_6_H_5_FeO_7_ (ferric citrate), 10^−3^ g L^−1^ of Na_2_EDTA·2H_2_O, 0.02 g L^−1^ of Na_2_CO_3_, 2.86 mg L^−1^ of H_3_BO_3_, 1.81 mg L^−1^ of MnCl_2_·4H_2_O, 0.222 mg L^−1^ of ZnSO_4_·7H_2_O, 0.390 mg L^−1^ of Na_2_MoO_4_·2H_2_O, 0.079 mg L^−1^ of CuSO_4_·5H_2_O, and 0.0494 mg L^−1^ of Co(NO_3_)_2_·6H_2_O. Algal cells were grown under continuous light intensity of 100 *μ*mol m^−2^ s^−1^ (SYLVANIA GRO-LUX Wide Spectrum light F40/GRQ/AQ/WS) at 24°C ± 1. Aliquots of algal samples were used for experiments when algal culture reached the exponential growth phase. The change of cell density was determined by monitoring the optical density at 750 nm, and the calculation was based on a standard correlation with the cell density measured using a multisizer Z3 (Beckman Coulter Inc., USA).

### 2.2. Synthesis of SPION

Superparamagnetic iron oxide nanoparticles (SPION) as Fe_3_O_4_ (SPION-1), Co_0.2_Zn_0.8_Fe_2_O_4_ (SPION-2), and Co_0.5_Zn_0.5_Fe_2_O_4_ (SPION-3) were produced according to the procedure reported in [11], by using the polyol process starting from Co(CH_3_CO_2_)_2_•4H_2_O, Zn (CH_3_CO_2_)_2_•2H_2_O and Fe(CH_3_CO_2_)_2_ as precursor salts and diethylene glycol as a solvent.

### 2.3. Stock Solution and SPION Characterization

In the preparation of stock solutions, SPION were suspended in culture medium at a concentration of 1 g L^−1^ and homogenized by ultrasonication during 30 min at 4°C to break up agglomerates. After sonication, stock solutions were mix with a vortex for 1 min, and various concentrations of SPION were prepared in culture medium for experiments. Size distribution was determined by dynamic light scattering (DLS) with a ZetaPlus particle sizer (Brookhaven Instrument Corporation, USA) using 90Plus Particles Sizing Software (Ver. 4.20). Zeta potential of SPION suspensions in culture medium was evaluated by the electrophoretic mobility method with the ZetaPlus system.

### 2.4. Experimental Treatments

In each treatment condition, the initial density of algal cells was of 10^6^ cells mL^−1^ in a final volume of 50 mL. Algal samples were exposed during 24, 48, and 72 h to 12.5, 25, 50, 100, 200, and 400 mg L^−1^ of SPION-1, SPION-2, or SPION-3, under the same illumination and temperature condition used for growing stock cultures. For the control sample, the same media composition was used but without any trace of SPION.

### 2.5. Growth Inhibition Test

The relative cell division rate (RCDR) was evaluated for 72 h according to [12], as RCDR = (ln⁡W_72 h_ − ln⁡W_0_)/72 h, where W_72 h_ represents cell density at 72 h and W_0_ the initial cell density.

### 2.6. Bioaccumulation of Fe, Co, and Zn

To separate SPION from cells, a sucrose gradient prepared in BG-11 media was done directly in a Beckman centrifuge tube inclined at a 30° angle in order to get 6 layers of different sucrose densities (5 mL of each sucrose solution 120, 100, 80, 60, 40, and 20%). Algal cells of control and SPION-treated samples were collected by centrifugation. Their pellets were slowly placed on top of sucrose gradient tubes which were centrifuged at 1,000 rpm during 30 min in a swinging-bucket 5810R centrifuge (Eppendorf, Germany). It resulted in the formation of a pellet of SPION at the bottom of the tube. The algal cells layer was recuperated with a glass Pasteur pipette and filtered on a 0.45 *μ*m filter previously dried and weighted. To remove SPION weakly bound to the cell surface or the filter, 3 × 10 mL of 10 mM ethylenediaminetetraacetic acid in BG-11 medium was slowly passed through the filter. Filters were dried at 95°C for 24 h and weighted to calculate algal dry weight. Filters were then placed in acid-washed glass tubes in which 4 mL of HNO_3_ and 500 *μ*L H_2_O_2_ were added. Samples were digested during 48 h at room temperature before being diluted to 20% HNO_3_ in Milli-Q purified water for the quantification of Fe, Co, and Zn using atomic absorption spectrometry analysis (Varian SpectrAA 220 FS, USA). Obtained Fe, Co, and Zn concentrations were normalized to the dry weight.

### 2.7. Soluble Fraction of Fe, Co, and Zn

Solubility of free Fe, Co, and Zn released from SPION suspensions were determined in culture medium. SPION suspensions was incubated during 24, 48, and 72 h in the same condition as described above for treatment. After incubation, NPs were removed by centrifugation at 12,000 g for 30 min and the supernatant was collected for analysis. Quantification of free Fe, Co, and Zn in solution was measured by atomic absorption spectrometry (Varian SpectrAA 220 FS, USA).

### 2.8. Production of ROS

The fluorescent dye 2′,7′-dichlorodihydrofluorescein diacetate ((H_2_DCFDA) Invitrogen Molecular Probe, USA) was used as an indicator of ROS according to [13]. Cellular esterases hydrolyze this probe into the nonfluorescent compound 2′,7′-dichlorodihydrofluorescein (H_2_DCF), which is better retained within cells. In the presence of ROS and cellular esterases, H_2_DCF is transformed into the highly fluorescent compound 2′,7′-dichlorofluorescein (DCF). H_2_DCFDA stock solution (10 mM) was prepared in ethanol in the dark. After 72 h of treatment, 1 mL of algal samples was exposed during 15 min to 0.2 mM of H_2_DCFDA in the dark. The ROS level was determined by measuring the fluorescence emission at 530 nm with a flow cytometer (FACScan system, Becton Dickinson Instruments, USA). Cytometry results were analysed using the WinMDI 2.8 software. Algal cells were separated from noncellular particles by using a relationship between particle size and red fluorescence level, originating from chlorophyll fluorescence emission. A positive control sample using methyl viologen was done to verify the assay (data not shown).

### 2.9. Cellular Viability

Viability of algal cells was estimated using the fluorescein diacetate ((FDA) Invitrogen Molecular Probe, USA) method according to [14]. FDA is a nonpolar ester compound which passes through cell membranes. Once inside the cell, FDA is hydrolyzed by esterases (enzymes present in viable cells) to produce fluorescein, accumulating in cell wall and emitting fluorescence under UV illumination. After 72 h of treatment, 1 mL of algal samples was exposed during 15 min to 0.2 mM of FDA in the dark. Cell viability was determined by measuring the fluorescence emission at 530 nm with a flow cytometer (FACScan System, Becton Dickinson Instruments, USA). Cytometry results were analysed using the WinMDI 2.8 software. Algal cells were separated from noncellular particles by using a relationship between particle size and red fluorescence level, originating from chlorophyll fluorescence emission. A positive control sample using methyl viologen was done to verify the assay (data not shown).

### 2.10. Chl *a* Fluorescence Emission

Photosynthetic electron transport was monitored from the change in the rapid rise of Chl *a* fluorescence emission using a “Plant Efficiency Analyser” fluorometer (Handy-PEA, Hansatech Ltd., UK). Total chlorophyll (Chl) content (*a* + *b*) was extracted in 100% methanol at 65°C and quantified with a spectrophotometer (Lambda 40, Perkin-Elmer, USA) according to the formula indicated in [15]: Total Chl (*μ*g mL^−1^) = (24.93 × A_652.4_ + 1.44 × A_665.2_).

Prior to fluorescence measurements, algal samples were transferred into clean sterile 2 mL glass vials and dark-adapted for 30 min. An aliquot of 5 *μ*g of total chlorophyll was gently filtered using low pressure filtration and algal cells were uniformly placed on a 13 mm glass fibre filter (Millipore, USA). The fluorescence induction was triggered using a 1 s saturating flash of 3500 *μ*mol m^−2^ s^−1^. The fluorescence intensity at 20 *μ*s was considered as the *O* value (*F*_*O*_); fluorescence intensities for *K*, *J*, and *I* transients were determined at 300 *μ*s (*F*_*K*_), 2 ms (*F*_*J*_), and 30 ms (*F*_*I*_), respectively. the maximum fluorescence yield reached maximal value of fluorescence intensity (*F*_*M*_) under saturating illumination. Different photosynthetic-based fluorescence parameters related to the functional state of Photosystem II were calculated [16, 17]: the maximum efficiency of PSII electron transport, *F*_*V*_/*F*_*M*_ = (*F*_*M*_ − *F*_*O*_)/*F*_*M*_; the absorption of photons by light harvesting antenna complexes (ABS) per active reaction center (RC), ABS/RC = ((*F*_*K*_ − *F*_*O*_)/0.25)×(1/(*F*_*J*_ − *F*_*O*_))×(*F*_*M*_/(*F*_*M*_ − *F*_*O*_)); the relative variable fluorescence yield at *J* transient, estimating the fraction of *Q*_*A*_ in its reduced state, *V*_*J*_ = (*F*_*J*_ − *F*_*O*_)/(*F*_*M*_ − *F*_*O*_); and the performance index of PSII photosynthetic activity, P.I. = RC/ABS × ((*F*_*M*_ − *F*_*O*_)/*F*_*O*_)×((1 − *V*_*J*_)/*V*_*J*_).

### 2.11. Statistical Analysis

All treatments were performed in triplicate. Means and standard deviations were calculated for each treatment. Significant differences between control and treated plants were determined by one-way analysis of variance (ANOVA) followed by a Dunnett's multiple comparison (DMC) test where *P* value less than 0.05 was considered significant.

## 3. Results

### 3.1. Characterisation of SPION

**Figure 1:**
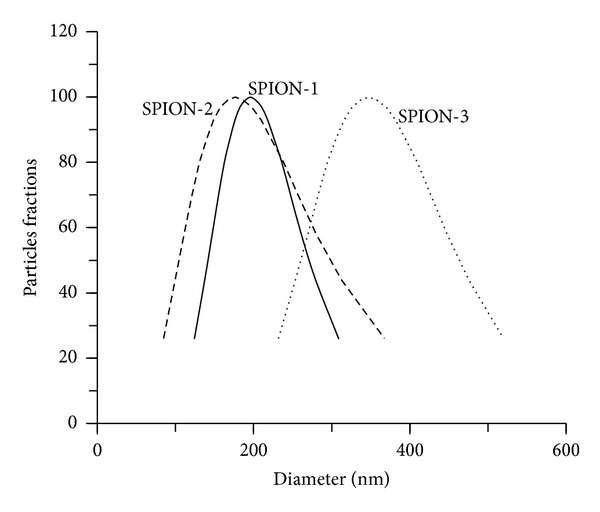
Particle size distribution of SPION-1 (Fe_3_O_4_), SPION-2 (Co_0.2_Zn_0.8_Fe_2_O_4_), and SPION-3 (Co_0.5_Zn_0.5_Fe_2_O_4_) suspensions in the culture medium.

When SPION were suspended in the media, nanoparticles formed agglomerates during the first minutes, as indicated by the distribution of hydrodynamic particles size diameter, which was caused by the content of salts in the media. Analysis by dynamic light scattering showed SPION-1, SPION-2, and SPION-3 suspensions in culture media to have an average diameter of particle size distribution of 195.9, 176.5, and 347.2 nm, respectively (Figure 1). These distributions of hydrodynamic size of SPION were found to be stable in the culture medium during the entire experimental exposure. Furthermore, measurements of zeta potential (mV) indicated that SPION were negatively charged in the media with values of −25.68 (±1.38), −29.14 (±3.85), and −28.06 (±1.19), respectively, for SPION-1, SPION-2, and SPION-3.

### 3.2. Solubility and Bioaccumulation of SPION

**Figure 2:**
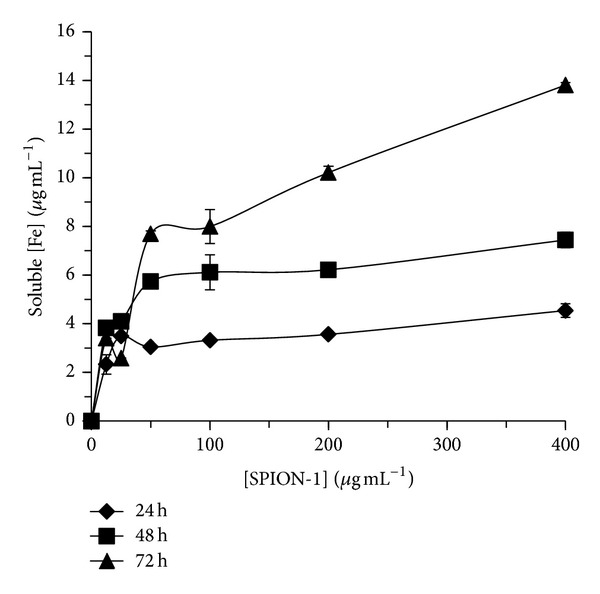
Soluble fraction of free Fe released from SPION-1 (Fe_3_O_4_) suspension in culture medium at 24, 48, and 72 h of exposure.

**Figure 3:**
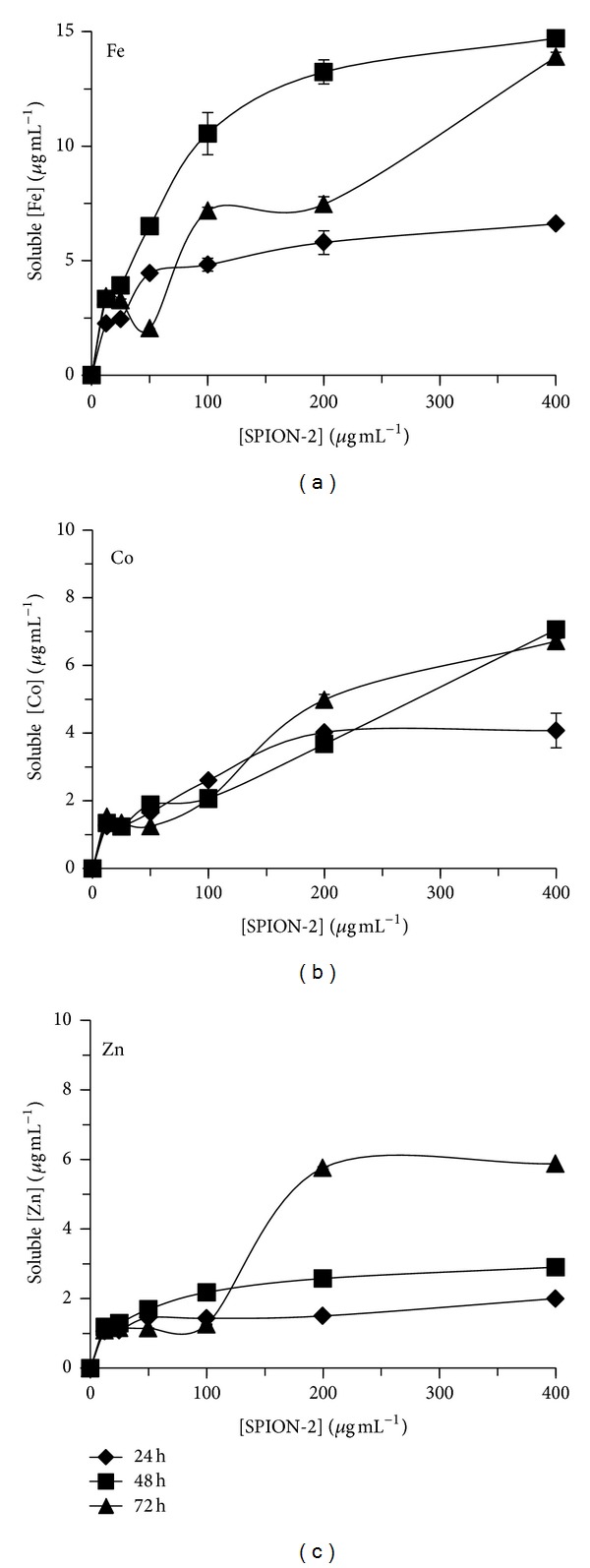
Soluble fraction of free Fe, Co, and Zn released from SPION-2 (Co_0.2_Zn_0.8_Fe_2_O_4_) suspension in culture medium at 24, 48, and 72 h of exposure.

**Figure 4:**
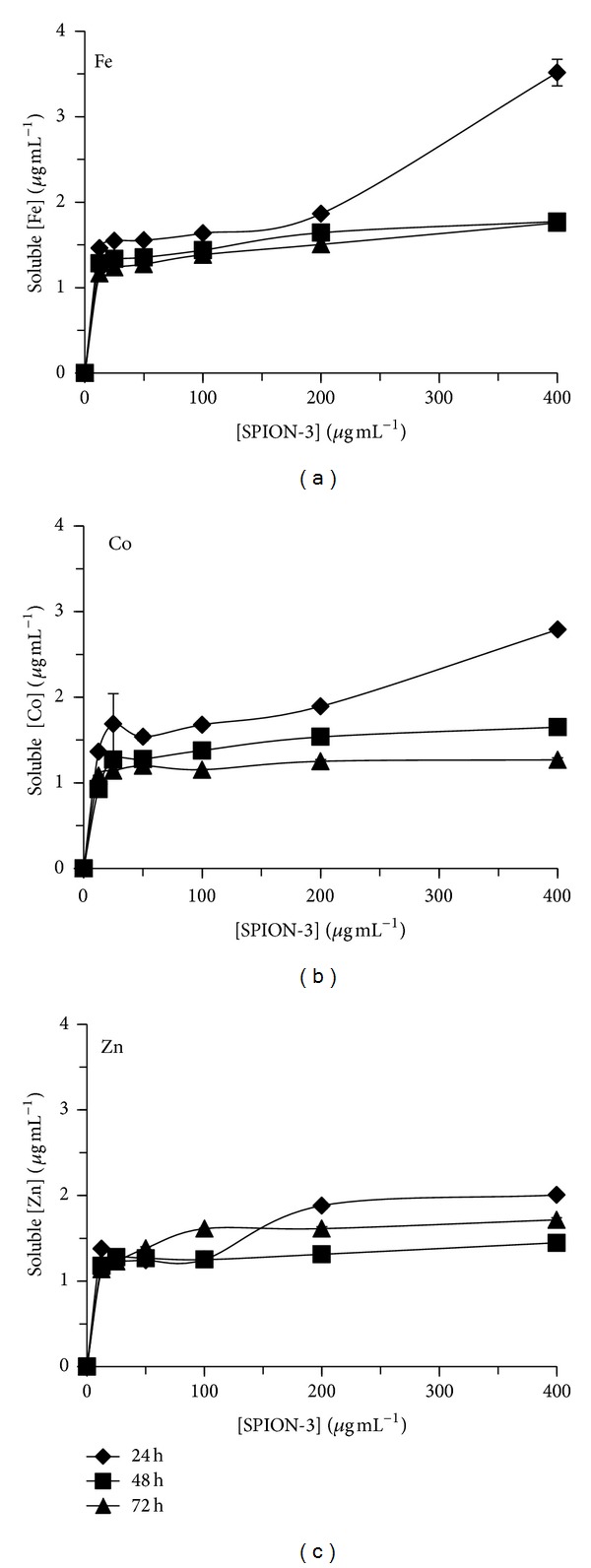
Soluble fraction of Fe, Co, and Zn released from SPION-3 (Co_0.5_Zn_0.5_Fe_2_O_4_) suspension in culture medium at 24, 48, and 72 h of exposure.

The soluble fraction of free Fe, Co, and Zn released from SPION-1, SPION-2, and SPION-3 suspensions in culture medium was determined at 24, 48, and 72 hours (Figures 2, 3, and 4). The quantity of soluble Fe, Co, and Zn in the medium was dependent on the time of exposure, the SPION composition, and their concentration. When *C. vulgaris* was exposed to 12.5 *μ*g mL^−1^ of SPION-1 (Fe_3_O_4_) during 72 h, the proportion of the soluble fraction of free Fe was of 27% compared to the nominal concentration of SPION-1, and it decreased to 3% for 400 *μ*g mL^−1^ of SPION-1 (Figure 2). However, the solubility of SPION-2 (Co_0.2_Zn_0.8_Fe_2_O_4_) was dependent on the metal species. When algal cells were exposed during 72 h to 12.5 *μ*g mL^−1^ of SPION-2, proportions of free Fe, Co, and Zn in the soluble fraction were, respectively, of 27, 12, and 9% compared to the nominal concentration of SPION-2 (Figure 3). For the exposure concentration of 400 *μ*g mL^−1^, the quantity of soluble Fe, Co, and Zn decreased to 3, 2, and 1%, respectively. Concerning the solubility of SPION-3 (Co_0.5_Zn_0.5_Fe_2_O_4_), concentrations of Fe, Co, and Zn in the soluble fraction at 72 h were of 9% each when compared to the nominal concentration of 12.5 *μ*g mL^−1^. For the exposure concentration of 400 *μ*g mL^−1^, concentrations of Fe, Co, and Zn in the soluble fraction were less than 0.5% in comparison to the nominal concentration of SPION-3. Indeed, we observed that the agglomeration of SPION increased their precipitation/sedimentation at the bottom of the experimental flask, which was directly related to the increasing concentration tested and time of exposure. This effect may cause the reduction of the surface contact of nanoparticles with the medium explaining the decrease in SPION solubilisation related to their concentration and time of exposure.

**Figure 5:**
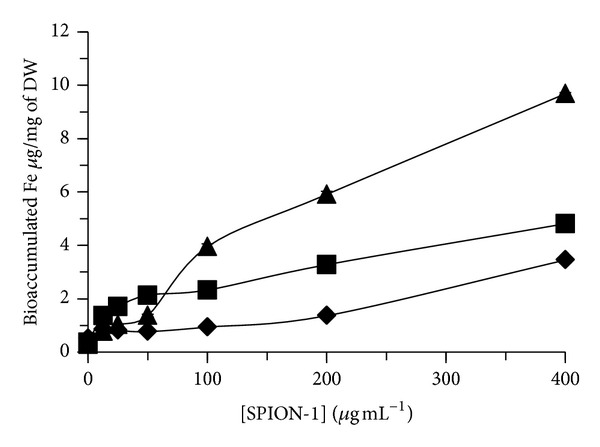
Bioaccumulated content of Fe in algal cells of *Chlorella vulgaris* exposed during 24 h (*◆*, diamond), 48 h (■, square), and 72 h (▲, triangle) to different concentrations of SPION-1 (Fe_3_O_4_).

**Figure 6:**
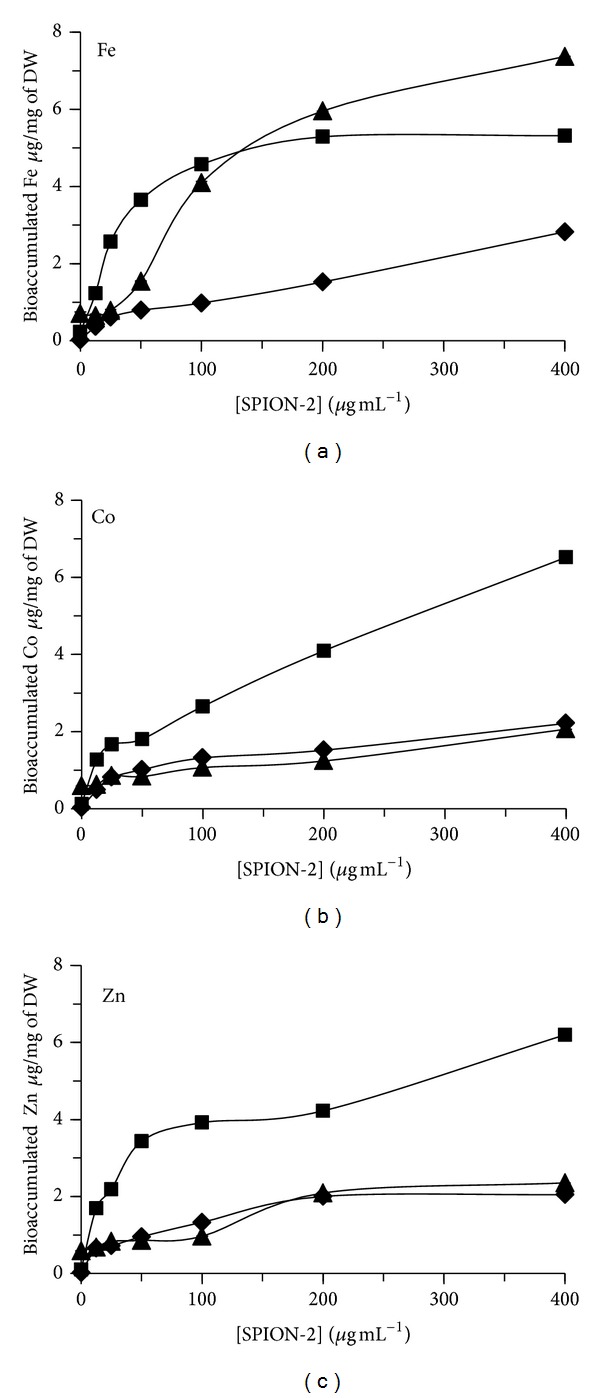
Bioaccumulated content of Fe, Co, and Zn in algal cells of *Chlorella vulgaris* exposed during 24 h (*◆*, diamond), 48 h (■, square), and 72 h (▲, triangle) to different concentrations of SPION-2 (Co_0.2_Zn_0.8_Fe_2_O_4_).

**Figure 7:**
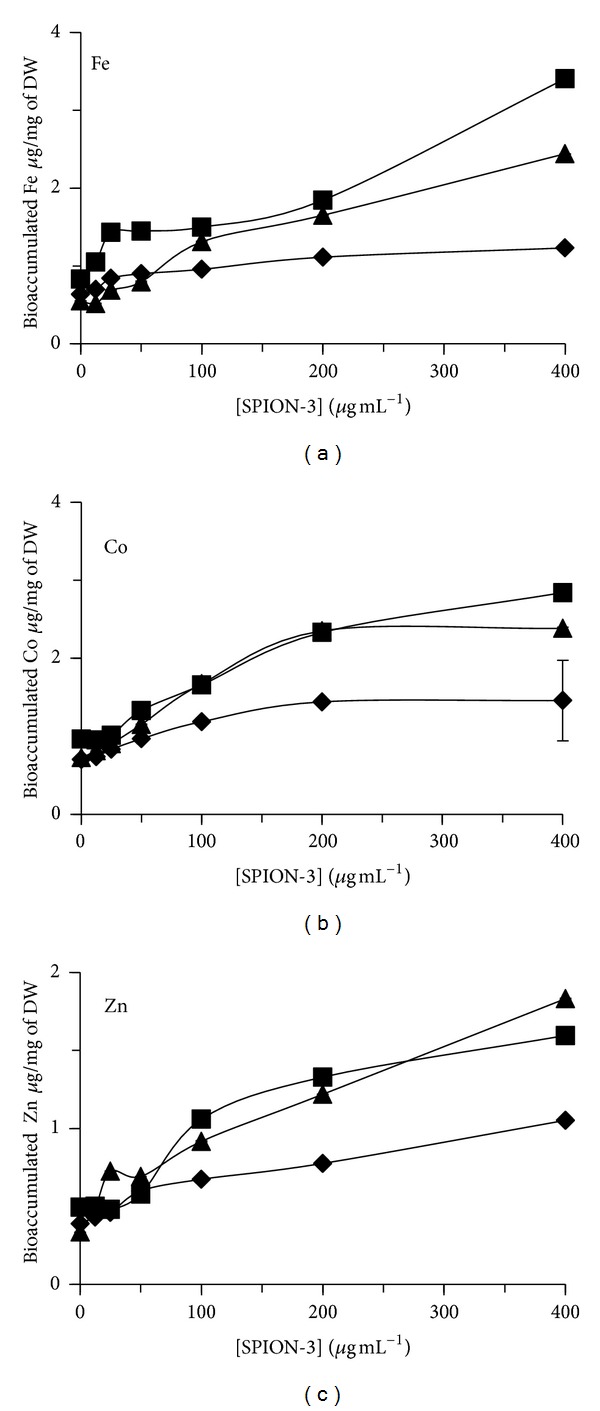
Bioaccumulated content of Fe, Co, and Zn in algal cells of *Chlorella vulgaris* exposed during 24 h (*◆*, diamond), 48 h (■, square), and 72 h (▲, triangle) to different concentrations of SPION-3 (Co_0.5_Zn_0.5_Fe_2_O_4_).

The bioaccumulation of total Fe, Co, and Zn was also quantified in algal biomass of *C. vulgaris* exposed during 24, 48, and 72 h to SPION (Figures 5, 6, and 7). For SPION-1, the accumulated contents of Fe in algal biomass increased in dependence on the concentration of SPION and the time of exposure (Figure 5). The bioaccumulated content of Fe increased by 35-fold for algal cells treated during 72 h to 400 *μ*g mL^−1^ of SPION-1 compared to control. Under this treatment condition, Fe bioaccumulation increased by 2.81-fold from 24 to 72 h. Concerning SPION-2, the accumulated contents of Fe, Co, and Zn increased in algal biomass in relation to the concentration of SPION (Figure 6). The content of Fe reached its maximum value at 72 h which increased by 10.4-fold for 400 *μ*g mL^−1^ of SPION compared to control. On the other hand, the contents of Co and Zn attained their maximal values at 48 h which increased, respectively, by 53.7-and 62.8-fold for 400 *μ*g mL^−1^ of SPION compared to control. When *C. vulgaris* was exposed to SPION-3, the bioaccumulation of Fe, Co, and Zn increased in relation to the concentration of SPION (Figure 7). At 72 h of exposure, values of Fe, Co, and Zn contents increased, respectively, by 4.42-, 3.29-, and 5.47-fold for 400 *μ*g mL^−1^ of SPION compared to control.

### 3.3. Inhibition of the Relative Cell Division Rate

When algal cells of *C. vulgaris* were exposed during 72 h to SPION at concentrations varying from 12.5 to 400 *μ*g mL^−1^, the inhibition of the relative cell division rate based on the change of cell density was dependent on the tested SPION concentration (Table 1). Under concentration exposure of 400 *μ*g mL^−1^, the relative cell division rate decreased significantly compared to the control by 47.8, 21.8, and 15.8% for SPION-1, SPION-2, and SPION-3, respectively.

### 3.4. Inhibition of Photosynthetic Electron Transport

**Figure 8:**
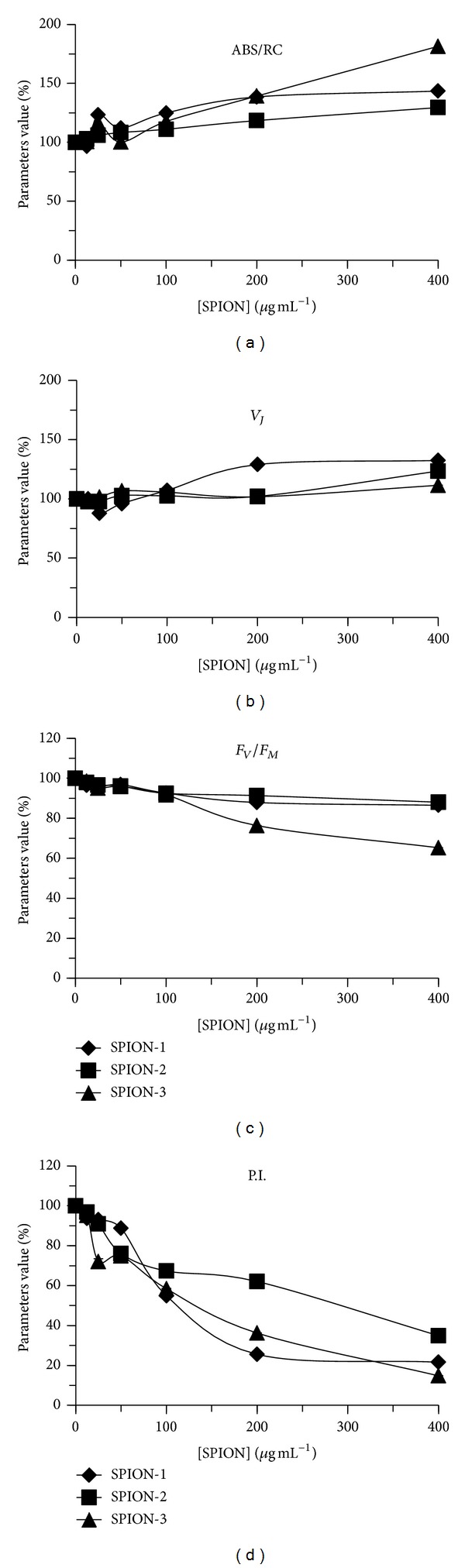
Change of different photosynthetic-based fluorescence parameters for algal cells of *Chlorella vulgaris* exposed during 72 h to different concentrations of SPION-1 (Fe_3_O_4_), SPION-2 (Co_0.2_Zn_0.8_Fe_2_O_4_), and SPION-3 (Co_0.5_Zn_0.5_Fe_2_O_4_). Maximal PSII quantum yield: *F*_*V*_/*F*_*M*_; ratio between the number of active PSII reaction centers and light harvesting Chl antenna size: ABS/RC; relative variable fluorescence yield at *J* transient: *V*_*J*_; performance index of PSII photochemical activity: P.I.

The change of Chl *a* fluorescence emission was used to monitor the photosynthetic electron transport when algal cells of *C. vulgaris* were exposed during 72 h to SPION toxicity. The kinetics of Chl *a* fluorescence indicated a decrease of fluorescence yields at *J*, *I*, and *M* levels without any change in their time of appearance, which was related to SPION concentration (See Supplementary Figures available online at http://dx.doi.org/10.1155/2013/647974). Furthermore, the change of photosynthetic-fluorescence parameters estimated from the fluorescence kinetic was used as biomarkers of SPION effect on PSII photochemistry (Figure 8). The strongest effect on photosynthetic electron transport was observed under 400 *μ*g mL^−1^ of SPION treatment. Under this condition, the value of ABS/RC parameter increased significantly compared to the control by 43, 29, and 81% for SPION-1, SPION-2, and SPION-3, respectively. This change indicated a decrease of functional PSII reaction centers able to perform photochemical reactions. Also, *V*_*J*_ values, indicating the level of Q_A_^−^/Q_A__(total)_, increased significantly compared to the control by 32, 23, and 12%, respectively, for SPION-1, SPION-2, and SPION-3, due to the inhibition of PSII electron transport flow toward to plastoquinone pool. However, the maximal quantum yield of PSII (*F*_*V*_/*F*_*M*_) decreased by 14, 12, and 35%, respectively, for SPION-1, SPION-2, and SPION-3. Moreover, the PSII performance index, P.I., was used as a global parameter integrating all PSII photochemical reactions from light harvesting energy transfer to electron transport. When algal cells of *C. vulgaris* were exposed during 72 h to 400 *μ*g mL^−1^ of SPION, P.I. values decreased significantly compared to the control by 78, 65, and 85% for SPION-1, SPION-2, and SPION-3, respectively.

### 3.5. Production of Reactive Oxygen Species Related to Cell Viability

**Figure 9:**
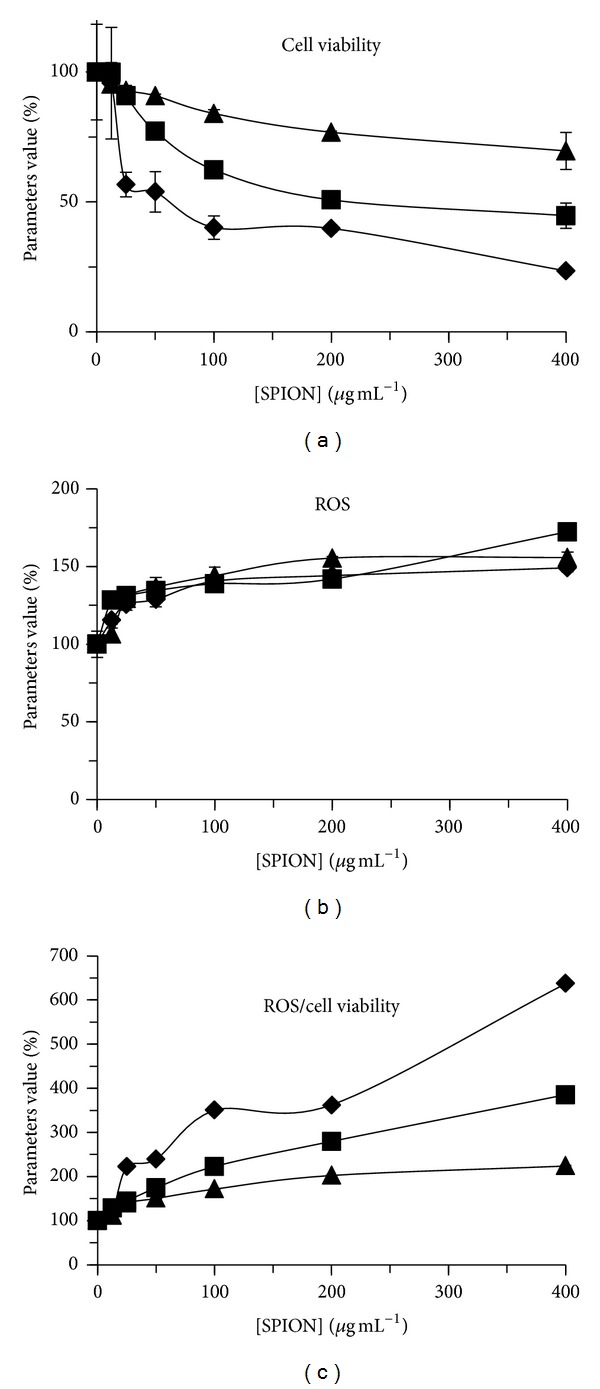
Change in the production of reactive oxygen species (ROS), cellular viability, and the ratio ROS/viable cells for *Chlorella vulgaris* exposed during 72 h to different concentrations of SPION-1 (*◆*, diamond), SPION-2 (■, square), and SPION-3 (▲, triangle).

The formation of ROS per viable cells was determined for *C. vulgaris* exposed during 72 h to SPION (Figure 9). Under these conditions, cell viability decreased significantly compared to control from 50 *μ*g mL^−1^ of SPION, indicating an induction of cellular oxidative stress. The production of ROS per viable cells increased significantly compared to control which was dependent on SPION species and concentration.

## 4. Discussion

### 4.1. Toxicity of SPION-1, SPION-2, and SPION-3

In this study, toxic effects of SPION-1, -2, and -3 were investigated on algal cells of *C. vulgaris*, which were caused by the deterioration of photochemical activities of photosynthesis, the induction of oxidative stress, and the inhibition of cell division rate. This complex cellular alteration was dependent on SPION chemical composition and its concentration in solution. Based on our results, it is evident that the bioaccumulation of free Fe, Co, and Zn from the soluble fraction was contributing to the toxicity impact in algal cells. It was previously proposed that the release of free metal ions from metallic nanoparticle suspensions represented a major source of toxicity for the growth rate of aquatic microorganisms [18, 19]. However, it is difficult to determine if the release of free metal ions from nanoparticles is the only contribution to the toxicity impact in the algal cellular system. Indeed, solubilisation of nanoparticles can take place either in the media or inside the cell, which possess an acidic pH environment favorable for particle solubility [20]. Therefore, another hypothesis is that SPION may also contribute directly to the toxicity impact. Recently, it was suggested for direct toxicity mechanisms of nanoparticles to induce a direct alteration of cellular exchanges with the media due to particles binding on cell membrane [21]. It was also shown for bioaccumulated Fe_3_O_4_ nanoparticles to cause direct toxic effects inside plant cells [8].

Moreover, the increase of production of reactive oxygen species per cell viability was related to the increasing exposure concentration of SPION, indicating that the induction of cellular oxidative stress was caused by the bioaccumulation of Fe, Co, and Zn. Indeed, it was shown in previous studies that metallic nanoparticles can cause the formation of ROS *via* the Fenton reaction or the disruption of major physiological processes [22, 23]. Also, the charged metallic surface of nanoparticles can trigger the formation of ROS by catalytic reduction of oxygen into superoxide anion, leading to oxidative damage into proteins, lipids, nucleic acids, and pigments [24].

When algal cells of *C. vulgaris* were exposed during 72 h to different concentrations of SPION suspensions, the relative cellular division rate was strongly inhibited for SPION-1 compared to SPION-2 and SPION-3. Under these treatment conditions, the production of ROS per viable cells was the highest for SPION-1. Indeed, Fe is well known to generate in cell free radical oxidations causing lipid peroxidation [25]. Furthermore, the change of fluorescence parameter P.I. indicated a stronger inhibition of photosynthesis for SPION-1. Therefore, according to the change of these biomarkers, the toxicity impact in algal cells of *C. vulgaris* was the strongest for SPION-1 suspensions.

### 4.2. Significance of Biochemical Biomarkers

In this study, two different biochemical biomarkers were used to characterize the toxicity of SPION: the production of ROS per viable cells and the photosynthetic-based fluorescence parameters. The change of parameter ROS/cell viability permitted determining the potential source of cellular toxicity causing the inhibition of cell division rate. On the other hand, fluorescence parameters related to PSII photochemical reactions have been shown to be sensitive biomarkers of SPION toxicity to *C. vulgaris* cells. Indeed, the Chl *a* fluorescence emission is demonstrated to be related to water-splitting system functions at PSII reaction center and to oxidoreduction states of electron transport carriers [26, 27]. Therefore, our results showed evidence for SPION to cause inhibitory effects on the PSII water-splitting system and the photoactivation of PSII reaction centers. In comparison to other fluorescence indicators, the performance index of PSII activity, representing an integrative indicator of all PSII photochemical reactions [17], was the most sensitive biomarker of the deterioration of PSII functions caused by SPION toxicity.

## 5. Conclusion

Nowadays, a large quantity of metallic nanoparticles is produced and their toxic potential as hazardous contaminants in aquatic environment needs to be investigated in order to develop specific bioassays for nanoparticles toxicity assessment. In this regard, microalgae represent sensitive organisms to be used in bioassay toxicity testing for the assessment of hazardous materials. In this toxicological investigation, we clearly showed the potential source of toxicity of three SPION suspensions, having different chemical compositions. The algal model *C. vulgaris *demonstrated to be a valuable bioindicator of SPION cellular toxicity which was indicated by the deterioration of photochemical activities of photosynthesis, the induction of oxidative stress, and the inhibition of cell division rate. Therefore, this work permitted characterising the cellular toxicity impact of these SPION with different biomarkers. A good understanding of these toxicological interactions will permit better understanding the risk of SPION toxicity for aquatic organisms.

## Supplementary Material

Supplementary Figure: Change in the Chl a fluorescence kinetics for algal cells of Chlorella vulgaris exposed during 72h to SPION-1 (Fe3O4), SPION-2 (Co0.2Zn0.8Fe2O4), and SPION-3 (Co0.5Zn0.5Fe2O4) at concentrations of 0 (a), 12.5 (b), 25 (c), 50 (d), 100 (e), 200 (f), 400 (g) *μ*g/mL.

## Figures and Tables

**Table 1:** Change in the relative cell division rate (RCDR) when *C. vulgaris* was exposed to SPION during 72 h.

[SPION] *µ*g/mL	RCDR (10^−6^)		
	SPION-1	SPION-2	SPION-3
0	0.335 ± 0.01	0.335 ± 0.01	0.335 ± 0.01
12.5	0.305 ± 0.05	0.307 ± 0.05	0.317 ± 0.004
25	0.308 ± 0.008	0.298 ± 0.006	0.318 ± 0.007
50	0.307 ± 0.02	0.297 ± 0.018	0.309 ± 0.004
100	0.266 ± 0.05	0.291 ± 0.007	0.306 ± 0.025
200	0.220 ± 0.008	0.264 ± 0.002	0.286 ± 0.005
400	0.175 ± 0.123	0.262 ± 0.0033	0.282 ± 0.004

## Abbreviations

Chl: Chlorophyll
NP: Nanoparticles
PEA: Plant Efficiency Analyzer
PSII: Photosystem II
ROS: Reactive oxygen species
SPION: Superparamagnetic iron oxide nanoparticles
TEM: Transmission electron microscope.
