# Supplementary figures and images for: Toxicity of Superparamagnetic Iron Oxide Nanoparticles on Green Alga Chlorella vulgaris
Source: Biomed Res Int. 2013 Dec 4;2013:647974. doi: 10.1155/2013/647974 (PMC3867878; doi:10.1155/2013/647974)

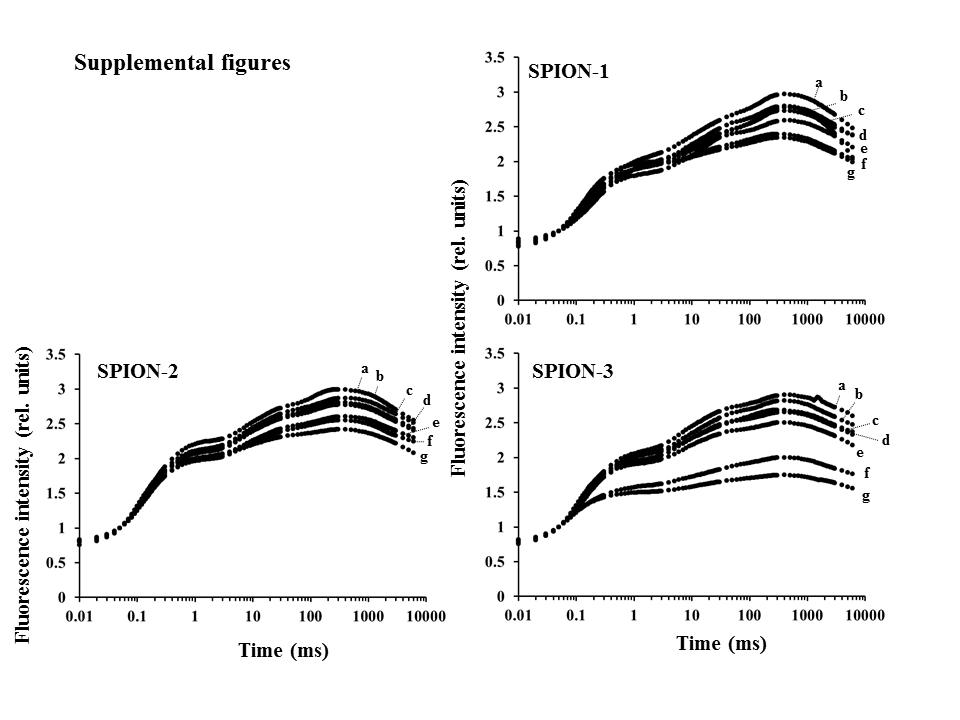

Supplement: Supplementary file 1 — Supplementary Figure: Change in the Chl a fluorescence kinetics for algal cells of Chlorella vulgaris exposed during 72h to SPION-1 (Fe3O4), SPION-2 (Co0.2Zn0.8Fe2O4), and SPION-3 (Co0.5Zn0.5Fe2O4) at concentrations of 0 (a), 12.5 (b), 25 (c), 50 (d), 100 (e), 200 (f), 400 (g) μg/mL. [file 647974.f1.tif]
